# Critical care delirium: prevention, identification and management: a narrative review

**DOI:** 10.1111/anae.70192

**Published:** 2026-03-11

**Authors:** Stephanie Kieswick, Ben Gibbison

**Affiliations:** ^1^ Department of Anaesthesia and Intensive Care University Hospitals Bristol and Weston NHS Foundation Trust; ^2^ Bristol Medical School University of Bristol Bristol UK

**Keywords:** delirium, intensive care

## Abstract

**Introduction:**

Delirium is a frequent complication of critical illness and remains an important cause of short‐ and long‐term morbidity for patients admitted to ICUs. Delirium is associated with prolonged mechanical ventilation; extended ICU and hospital stay; and longer‐term health issues. Development is associated with patient (e.g. severe physiological derangement); clinical (e.g. sedation); and environmental factors (e.g. loss of day/night variation and sleep deprivation). This review provides an overview of the current understanding of ICU delirium and its implications for critical care practice.

**Methods:**

We undertook a narrative review of the contemporary literature and synthesised evidence related to epidemiology, pathophysiology, risk factors, diagnostic tools and preventive and therapeutic strategies, with an aim of developing a practical resource for clinicians.

**Results:**

Delirium impacts approximately one‐third of patients admitted to general ICUs, with higher rates among older adults and those requiring mechanical ventilation. Diagnosis relies on clinical assessment supported by validated instruments, each with limitations for the critically ill population. Pharmacological interventions have not shown consistent benefit to prevent or treat delirium. In contrast, multicomponent non‐pharmacological approaches (e.g. optimal sedation, early mobilisation, re‐orientation, sleep hygiene and family engagement) are associated with a reduced incidence of delirium and improved functional outcomes. Delirium contributes to the long‐term psychological and cognitive burden of critical illness and structured follow‐up and ICU diaries may support recovery.

**Discussion:**

Delirium in the ICU is common and important for patients and multidisciplinary critical care providers. The most effective strategies for prevention and management are non‐pharmacological and require co‐ordinated, multidisciplinary delivery. Sustained improvements in outcomes require consistent implementation of evidence‐based care bundles and better integration of follow‐up services for survivors.

## Plain Language Summary

**What we did?:**

Delirium is when people become confused or see things that are not real when they are very sick, common in intensive care units. We read studies, clinical trials, and medical guidelines to explain what delirium is, why it happens, how doctors and nurses identify it and what helps prevent or treat it. Our goal was to bring all this information together in a summary so that healthcare staff can better care for patients.

**Why did we do it?:**

Delirium happens to many people in intensive care units and can be very upsetting for both patients and their families. It can also slow down recovery and make people stay in hospital longer and cause problems with memory and thinking after they go home. We wanted to explain why delirium happens and tell healthcare staff the best ways to prevent it and help people recover.

**What we found?:**

We explain that delirium is very common in ICU, affecting about one out of every three patients, and even more in older people. Medicines have not been very good at stopping delirium. Instead, simple things help the most, like helping patients sleep, getting them moving as soon as possible, reminding them where they are, letting family visit, and using less strong sedation. These things work best when doctors, nurses, and therapists all work together. We also suggest that people may need support after leaving ICU to help them understand and recover from their delirium experience.

## Introduction

Delirium is a common and serious complication for patients with critical illness, affecting up to 70% of those admitted to ICU [1]. As populations age and the number of patients with multiple chronic conditions who require intensive care continues to rise, the incidence of ICU delirium is expected to increase. Clinically, ICU delirium is characterised by acute disturbances in attention, awareness and cognition, often accompanied by hallucinations, disorganised thinking and profound confusion [2]. These symptoms are distressing for patients and their families and present a significant challenge for ICU staff.

The presence of delirium in patients who are critically ill is associated with substantially worse outcomes. Compared with patients without delirium, those affected experience more than a two‐fold increase in both in‐hospital and 6‐month mortality [3, 4, 5, 6], and have an extended duration of hospital stay (largely within the ICU) [3, 6, 7]. The mechanism behind worse outcomes is thought to be impaired communication and treatment adherence, creating a cycle of prolonged mechanical ventilation; extended ICU stay; physical deconditioning; and greater risk of complications. Beyond the acute phase, patients who develop ICU delirium are more likely to require hospital or emergency readmission [8], and are at increased risk of long‐term cognitive decline [9], dementia and post‐traumatic stress disorder [10, 11, 12, 13].

Whilst delirium is prevalent in all acute care settings, there are some features of delirium in the ICU that make its identification, prevention and management different to other areas. The severity of acute illness and need for interventions increases the risk of delirium for a wider age‐group (up to 70% of all admissions in some cohorts [1]) than in other acute settings, where delirium mainly affects older adults (around 30% of adults aged ≥ 65 y [14]). In addition, sedative drugs given to patients to tolerate mechanical ventilation can both mask and compound delirium. Further, the ICU environment is noisy day and night, and patients may have multiple machines (e.g. ventilator, renal replacement therapy) and monitoring/therapeutic catheters attached to them. These factors limit sleep, which further compounds delirium.

This review aims to give an overview of the key factors for delirium, including diagnosis, prevention and management, but also to discuss potential translation into improved clinical practice.

## Methods

We undertook a narrative review of the contemporary literature and synthesised evidence related to epidemiology, pathophysiology, risk factors, diagnostic tools and preventive and therapeutic strategies, with the aim of developing a practical resource for clinicians. Information about diagnostic tools, preventative and therapeutic strategies were drawn from previously published reviews performed as part of the OPTIC Project (NIHR204591) [15, 16, 17, 18] – an NIHR funded project focused on establishing the optimal way to prevent and manage ICU delirium.

## Results

Delirium is one of the few clinical diagnoses made in the ICU – confirmed purely by history and examination. There are no investigations (e.g. blood tests) that have been shown to diagnose delirium reliably. Diagnosis is confirmed by fulfilling the criteria of the DSM‐5 (Diagnostic and Statistical Manual of Diseases, 5th Edition [2]; Box 1).

Box 1 **The DSM‐5 criteria for delirium [2].**

**A**. A disturbance in attention (i.e. reduced ability to direct, focus, sustain and shift attention) and awareness (reduced orientation to the environment).

**B**. The disturbance develops over a short period of time (usually hours to a few days), represents a change from baseline attention and awareness and tends to fluctuate in severity during the course of a day.

**C**. An additional disturbance in cognition (e.g. memory deficit, disorientation, language, visuospatial ability or perception).

**D**. The disturbances in criteria A and C are not explained by another pre‐existing, established or evolving neurocognitive disorder and do not occur in the context of a severely reduced level of arousal, such as coma.

**E**. There is evidence from the history, physical examination or laboratory findings that the disturbance is a direct physiological consequence of another medical condition, substance intoxication or withdrawal (i.e. due to a drug of abuse or to a medication), or exposure to a toxin, or is due to multiple aetiologies.

Beyond this, delirium is generally classified into three motoric subtypes according to clinical phenotype (Box 2) [19]. Hyperactive delirium is typically the easiest subtype to recognise but least prevalent; whereas hypoactive delirium is the most prevalent, associated with the poorest outcomes, but is the least recognised [20]. Work is continuing to find and define other sub‐types of delirium, either by risk factors, symptoms, precipitants, mechanism or other features – but further research is needed to validate these phenotypes and define their relevance for clinical care [21].

Box 2 **The different motoric subtypes of delirium.**

- **Hypoactive delirium** presents with reduced motor activity, lethargy, diminished arousal and emotional withdrawal.
- **Hyperactive delirium** is characterised by increased motor activity, agitation and restlessness. Patients may be combative or aggressive.
- **Mixed delirium** exhibits alternating features of both, often fluctuating over the course of the day.

### Incidence

The incidence of delirium in adult patients on the ICU varies from 20% to 80%, dependent on the case‐mix and assessment tools. Large multicentre, prospective studies and systematic reviews have documented pooled incidence rates of 26–32% in general ICU cohorts, with substantially higher rates (up to 75%) among patients whose lungs are mechanically ventilated and older adults [3, 20].

The prevalence of delirium during the ICU stay is similarly heterogeneous. The DECCA study, a one‐day‐point prevalence study of 497 patients in 104 ICUs from 11 countries including the Americas and Spain, found a pooled delirium prevalence of 32.3% [1]. Similarly, a systematic review including centres from Europe, Asia and the Americas (27,342 patients), found a pooled prevalence of 31.8% [20]. This close agreement in prevalence using studies of different methodologies suggests these estimates are likely generalisable. Higher prevalence rates are observed in specific subgroups, such as neurological conditions or very severe illness.

The relative distribution of delirium motoric subtypes varies with case‐mix. In the general ICU population, hypoactive delirium predominates, whereas mixed delirium is more common in certain subgroups, such as postsurgical patients. Overall, hypoactive delirium accounts for approximately half of ICU delirium cases, with mixed and hyperactive subtypes making up the other half comprising 15–25% each [20, 22].

### Short‐ and long‐term effects of ICU delirium

Delirium in the ICU is strongly associated with adverse outcomes, including prolonged duration of ICU and hospital stay; increased healthcare costs; and increased short‐term mortality. Evidence from meta‐analyses and large cohort studies consistently indicates that delirium in patients who are critically ill roughly doubles the risk of in‐hospital mortality compared with patients without delirium (risk ratio 1.7–3.2) [3, 4, 5, 6]. Excess mortality risk appears most pronounced during the ICU stay itself, particularly in the first days following delirium onset, and is greater in patients with persistent delirium than in those with rapidly reversible (often sedation‐related) delirium [3, 6, 7]. The risk of death increases with the duration of delirium, with each additional day of delirium associated with greater risk [23].

Once time‐variance confounders and competing risks (such as coma) are taken into account, there appears to be little direct contribution of delirium to in‐hospital mortality (< 1% attributable risk [4, 24]). The observed link with short‐term mortality may instead reflect the underlying disease severity as a confounder i.e. the underlying disease severity affects the risk of delirium and mortality. Delirium may affect mortality, but the data show that this effect is small [4, 24]. Failure to control for underlying disease severity leads to an overestimation of the risk of delirium affecting mortality. Days spent in a coma are more strongly associated with short‐term mortality than days spent with delirium; and many studies use delirium‐ and coma‐free days as their outcome, leading to further unmeasured confounding. Nevertheless, delirium remains a clear marker of poor prognosis and adverse short‐term outcomes in the ICU.

Delirium independently increases duration of stay (in the ICU and in‐hospital) and this is a major driver of the increased healthcare costs [25, 26, 27]. The duration of the delirium is directly proportional to the duration of stay [28, 29]. Each day of delirium adds over a day to ICU stay [5, 30]. This increased duration of stay is thought to arise from prolonged duration of mechanical ventilation; higher rates of complications (such as unplanned removal of tracheal tubes and catheters); and failure to engage in medical management, weaning and rehabilitation.

Delirium in the ICU has not been consistently shown to be independently associated with long‐term (post‐discharge) mortality risk [31]. Patients who develop delirium in the ICU have about twice the risk of longer‐term (months to years) mortality (hazard ratio 1.4–2.2) [30, 32, 33]. The risk is most pronounced in the first 30 days to 1‐year post‐discharge. However, this association is subject to similar confounders (e.g. disease severity) as short‐term mortality. So, whilst delirium is a robust marker of mortality after ICU and hospital discharge, it is probably not the cause of it.

Delirium in the ICU is an important factor in cognitive dysfunction after discharge, with deficits similar to mild Alzheimer's disease or moderate traumatic brain injury [9]. Up to 70% of ICU patients with delirium show significant cognitive impairment up to 18 months after discharge [34]. These deficits occur in patients of all ages (not just older adults) and persist with only slight improvement [9]. The duration of delirium is the most important predictor of this cognitive dysfunction [9], persistent even after severity of illness is controlled for. However, the actual contribution of delirium to cognitive dysfunction has not been quantified.

Delirium is postulated to be a causal factor for depression, post‐traumatic stress disorder and poor quality‐of‐life, yet there is little evidence to support this [31]. Multiple studies have shown that ICU survivors who experience delirium are at increased risk of subsequent psychiatric morbidity, including depression, anxiety and post‐traumatic stress disorder symptoms, although the strength of these associations varies across studies [10, 11, 12, 13]. Approximately one‐third of patients develop clinically significant depression [11], around one‐quarter develop post‐traumatic stress disorder [13] and just under a half report anxiety [10, 13] within 1 year of discharge. The development of these conditions reflects a complex interplay of risk factors, including pre‐existing mental health disorders; acute psychological stress during critical illness; and physical impairment after ICU stay. Whilst delirium may contribute to these outcomes, it is unclear to what extent.

### Pathophysiology

The pathophysiology of delirium is complex, multifactorial and incompletely understood. This reflects the interaction between several pathophysiological processes and the vulnerability of the underlying brain. Both critical illness (e.g. sepsis) and ICU care (e.g. sedation, sleep deprivation) have been implicated in causing neuroinflammation and oxidative stress, alongside neurotransmitter imbalance (e.g. acetylcholine deficiency and dopaminergic excess), blood–brain barrier dysfunction and impaired cerebral perfusion [35, 36].

At a systems level, neuroimaging studies suggest that delirium is associated with reduced cerebral metabolism and disrupted connectivity within networks related to attention, arousal and introspection [37, 38]. These observations are corroborated by EEG studies, which consistently show increased proportions of δ and θ activity in patients with delirium compared with healthy, awake volunteers. Such slowing of brain activity may reflect aberrant cortical activity in regions responsible for arousal regulation, perception, attention and higher‐order cognitive processing [39]. Ultimately, patients with delirium exhibit structural changes on brain imaging including cerebral atrophy; volume loss; and white matter lesions [38, 40]. These are, however, not uniformly permanent. While prolonged delirium is associated with persistent cerebral atrophy (and cognitive decline), the white matter changes may reflect pre‐existing pathology or potentially reversible acute brain injury, rather than irreversible structural damage directly caused by delirium.

The interaction between the pathophysiological processes and the underlying vulnerability of the brain manifest in the clinical risk factors for ICU delirium [41]. These can be categorised into brain vulnerability; the impact of the critical illness; and the impact of the ICU care (Table 1). It is noticeable that at the point of ICU admission, those risk factors causing brain vulnerability are largely non‐modifiable and those in the impact of ICU care are all modifiable, with some of the impact of critical illness partially modifiable. This highlights that prevention and management of ICU delirium should be directed toward mitigation of the critical illness and modification of ICU care. It is beyond the scope of this article to go into all the risk factors in detail. However, some are worth highlighting.

**Table 1:** The clinical risk factors for ICU delirium.

Brain vulnerability	Impact of critical illness	Impact of ICU care
Increasing age	Sepsis	Sedation type
Pre‐existing cognitive impairment	Multiorgan failure	Mechanical ventilation
Pre‐existing psychiatric illness	Hypoxaemia	Sleep disruption (including lack of day/night variation)
Pre‐existing neurological disease (e.g. stroke)	Metabolic derangements (e.g. electrolyte imbalance, renal/hepatic failure)	Drugs (e.g. anticholinergics, opioids)
Substance or alcohol misuse	Shock/impaired cerebral perfusion	Sensory deprivation or overload (e.g. lack of stimulation during day/noise at night)
Sensory impairment (hearing/ vision loss)	Systemic inflammation	Reduced activity
Genetic predisposition (e.g. *APOE ε4 allele*)	Pain	

Age is strongly associated with higher risk, incidence, severity and duration of delirium in ICU patients [42]. The odds of developing delirium increase with each additional year of age, and patients aged over 65 years have a markedly higher incidence compared with younger adults [43]. Advanced age amplifies vulnerability to delirium in the presence of critical illness exposures such as organ failure; mechanical ventilation; and illness severity. Dementia and frailty further increase this risk, with delirium triggered at a lower threshold.

The *APOE ε4* allele is the most significant known genetic risk factor for Alzheimer's disease. Presence of this gene is also associated with twice the risk of experiencing delirium in the ICU and a longer duration of stay if it occurs [44, 45]. This risk is present even when no outward signs and symptoms of Alzheimer's disease are seen [44, 45].

The type and depth of sedation used impacts the risk of delirium. Benzodiazepines clearly increase the risk of delirium by around one‐third compared with propofol [31, 46] and should be avoided. Propofol is probably an intermediate risk for delirium compared with other sedatives [31].

Disentanglement between the interrelated factors of sleep, physical activity, circadian rhythms and delirium is difficult. The intrinsic human circadian rhythm is typically slightly longer than 24 h but is entrained to the day‐night cycle by cues such as light–dark exposure; physical activity; and meal timing [47]. Critical illness and its management disrupt these mechanisms and circadian hormone patterns (e.g. cortisol secretion) are blunted [48]. Furthermore, there is frequently continuous artificial lighting and a lack of daylight in ICU environments. Physical and cognitive activity is minimal, and nutrition is frequently delivered continuously via gastric feeding tubes. These all reduce the likelihood of night‐time sleep, and delirium itself further impairs sleep–wake regulation. There is a paucity of mechanistic data that directly links these processes to delirium. However, these factors are all modifiable, with interventions at low risk of harm and likely to be inexpensive.

Acetylcholine plays a role in memory and cognition. Low concentrations in the brain have been implicated mechanistically in delirium development [49], but robust evidence for this is low, as measuring brain acetylcholine levels in a live person is challenging [50]. Drugs that have centrally acting anticholinergic effects are thought to increase the risk of delirium, and there is some weak evidence for this [51, 52]. Opioid withdrawal after medium‐ and long‐term sedation is a candidate for precipitating delirium [53] but there is as yet little or no evidence to support structured weaning programmes to prevent delirium [31].

### Diagnosis

Delirium is diagnosed only on clinical grounds – consequently, most non‐psychiatric clinicians rely on structured tools and instruments to support them in diagnosis. These are essentially standardised methods of applying DSM‐5 or other diagnostic criteria, and more than 20 have been developed to specifically identify and measure ICU delirium.

The distinction between a tool and an instrument lies in validation. Instruments are validated scientifically and usually standardised, whereas tools are often not. This difference is important, as the validity of an instrument determines its proper use case. Screening instruments are designed for broad application: they have high sensitivity but low specificity. Diagnostic instruments, by contrast, have high specificity but lower sensitivity. Despite this distinction, there remains considerable confusion (both in the literature and in practice) about which tools or instruments are appropriate in different contexts. For example, the Confusion Assessment Method for the ICU (CAM‐ICU) [54] was developed and validated as a diagnostic tool but is used frequently (and recommended) as a screening tool [55]. While this may appear to be a minor issue, it is not: the CAM‐ICU provides only a snapshot at a single point in time. Even if applied twice daily, it may still fail to capture the fluctuating course of delirium.

The most widely used instruments for assessing ICU delirium are the CAM‐ICU [54], the Intensive Care Delirium Screening Checklist (ICDSC) [56] and the 4AT [57]. These are applied in clinical practice and as outcome measures in research trials. However, implementation has faced challenges and these instruments are not currently included in national audits of intensive care quality and outcomes (e.g. Intensive Care National Audit and Research Centre). The CAM‐ICU is the most used and cited instrument for diagnosing delirium in intensive care. It is applied for clinical screening, diagnosis, audit and as an outcome measure in drug trials with original validation based on a cohort of 96 patients at a single centre in the USA [54]. The assessment involves two steps: first, determine level of consciousness using the Richmond Agitation‐Sedation Scale; and then, if the patient is sufficiently awake, completion of tasks mapped to DSM‐IV delirium domains. In 2014, the CAM‐ICU was updated to align with DSM‐5 criteria [58] and a related tool, the CAM‐ICU‐7 [59], was developed to provide a severity score for delirium. The ICDSC is an eight‐item checklist. Whilst this accounts for the fluctuating nature of delirium (by covering a time‐period rather than a single point), it is generally reported to be less sensitive and specific than the CAM‐ICU and is therefore used less commonly. It also places a heavy burden on assessors (usually the ICU nurse) because several items require considerable subjective judgement. For example, one domain asks whether the patient shows “*gross impairment in reality testing*” but offers no clear guidance on how to define or assess this, making systematic application difficult in routine practice. The 4AT is a four‐question screening tool originally developed for general hospital patients rather than ICU settings. It is quick, objective, requires minimal training and has shown sensitivities and specificities of about 85% in large, multicentre international studies [57]. However, two of its items require verbal interaction with the patient, which makes it impractical in ICUs where many patients tracheas are intubated. This is a major limitation, as approximately half of UK ICU patients require ventilation during their ICU stay.

### Prevention and management

It is estimated that 30–40% of delirium is potentially preventable [35]. More intensive, targeted preventative strategies are warranted for those at higher risk (e.g. older adults and patients whose lungs are mechanically ventilated) and secondary prevention is appropriate for patients who already have delirium. There is considerable overlap between prevention and management, since many strategies used in treating delirium also serve as secondary prevention. Approaches can be broadly divided into pharmacological and non‐pharmacological interventions.

There is no robust evidence to support the routine use of pharmacological strategies for the prevention of delirium, nor is there strong evidence to guide pharmacological management once delirium has developed [15]. A range of pharmacological approaches have been investigated, targeting different proposed pathophysiological mechanisms [15]. These include the use of alternative sedation regimens; antipsychotic drugs; anticholinesterase drugs; corticosteroids; and sleep‐promoting medications. However, none have shown consistent efficacy (despite their inclusion in some guidelines) in either prevention or treatment.

The type of sedation used, as well as the depth of sedation, impacts the risk of delirium. Propofol is probably at an intermediate risk of delirium compared with other sedative drugs [31]. Benzodiazepines (particularly midazolam) increase the risk of delirium by around one‐third compared with propofol [31, 46] and so are not recommended [55]. The place of dexmedetomidine is more controversial. This has some qualities (such as better patient communication), which make it attractive as a sedative drug that may reduce delirium, and indeed it has previously been widely held to reduce the risk of delirium when used as a sedative drug. It has consistently shown reductions in the incidence of delirium (by about a third), duration of mechanical ventilation (by about a day) and duration of stay (by about a day) [31, 60]. However, this information was based on tightly controlled efficacy trials. When applied in pragmatic, clinical effectiveness trials, serious episodes of bradycardia and agitation have been observed [61, 62]. There was also excess mortality with higher doses of dexmedetomidine in younger patients [63]. However, again, this may have illness severity as a confounder. Subsequently, the 2025 update of the guidelines of Society of Critical Care Medicine gives a new conditional recommendation for dexmedetomidine over propofol in adults whose lungs mechanically ventilated, where light sedation and/or a reduction in delirium are the highest priority.

Sedation breaks (daily sedation interruption) and sedation titration to light levels are safe and effective strategies to prevent ICU delirium (especially when combined) [64]. Some of this may, in part be due to increased arousal and mobility, as well as the reduced deliriogenic effect of the sedation. The certainty of sedation interruption and targeting to reduce delirium is very low [65]. Nevertheless, targeting reduced exposure to sedation is included in all guidelines for the prevention and management of ICU delirium [31, 55].

There is no evidence that the prophylactic administration of antipsychotics (e.g. haloperidol, risperidone, quetiapine) reduces the incidence of delirium in the ICU [15]. Similarly, once delirium is established, the available evidence provides little support for antipsychotics in reducing its duration or severity [31, 66]. Their principal role remains the short‐term management of agitation, with the aim of preventing patients from harming themselves or others. Importantly, antipsychotics may be harmful in patients with hypoactive delirium, in whom agitation is not a feature.

Although quetiapine has become a popular option for managing agitation in the ICU, current evidence indicates that it is not more effective than haloperidol [66, 67]. Multiple randomised controlled trials and comparative studies have shown no significant differences between quetiapine and haloperidol in terms of delirium resolution or agitation control [66]. However, patients treated with haloperidol appear to have more than twice the risk of developing extrapyramidal adverse effects [66, 67].

As sleep deprivation is considered a modifiable risk factor for delirium, this is an attractive target for intervention [68, 69]. Multiple drugs that are aimed at promoting sleep, such as melatonin and ramelteon (a melatonin receptor agonist), have been studied to prevent and manage delirium. Melatonin is prescribed commonly in the ICU to aid with sleep due to the low risk of adverse effects. The latest update to the Society of Critical Care Medicine guidelines recommends melatonin in adult patients to reduce delirium, but the evidence for this is of low certainty [68]. There is no specific recommendation regarding dose, duration and frequency due to the vast heterogeneity of the 34 included trials. Ramelteon has also been trialled and may be effective [15], but its use is less widespread. Non‐benzodiazepines (e.g. zopiclone) are sedatives and therefore may precipitate delirium and so should be avoided in this setting [31].

It has been suggested that corticosteroids can cause and treat delirium. Both suggestions came from observational and cohort studies [70] when illness severity could not be controlled for. However, in large prospective studies [71, 72] and meta‐analyses [15] there appears to be little association between corticosteroid administration and any neuropsychiatric symptoms, including delirium. Whilst anticholinesterase drugs make an appealing intervention to prevent and manage ICU delirium (given the biological mechanism and their effectiveness for treatment of dementia) there is little evidence supporting their use, and they may be associated with harm [73, 74].

Non‐pharmacological interventions are inexpensive and have a low risk of adverse effects [17]; however, they are difficult to implement for multiple reasons. There is wide heterogeny in the definitions of non‐pharmacological interventions which makes evidence synthesis difficult. It is easy to prescribe a drug at a set dose and frequency. It is potentially more difficult to define what ‘early mobilisation’ is at different stages of the care pathway and in different ICUs. Non‐pharmacological interventions fall broadly into four categories [17]: family; environmental; communication; and activity/mobilisation interventions (Box 3). There is little robust evidence for any of these interventions except re‐orientation using the family voice and early mobilisation [17].

Box 3 **Four categories of non‐pharmacological interventions to prevent delirium.**

- **Family interventions**

These include re‐orientation using the family voice, family visiting and family photos by the patient's bedside.

- **Environmental interventions**

These include bright light during the day and maintaining darkness at night and the use of eye masks and ear plugs.

- **Communication interventions**

This includes re‐orientation by staff and a structured multidisciplinary assessment and care.

- **Activity/mobilisation interventions**

This includes physiotherapy; occupational therapy; early mobilisation; and cognitive exercises. The definition of early mobilisation is variable, but mobilisation within 72 h of ICU admission is easily operationalised and achievable in many patients.

### Delirium care packages

There are many guidelines for the prevention and management of delirium in acute care. Most of these are not designed for the ICU setting (including current National Institute for Health and Care Excellence guidance [75]). There are three national and international guidelines that are relevant; multicomponent non‐pharmacological care is the cornerstone of them. *Guidelines for the Provision of Intensive Care Services* [76] defines the planning and delivery of UK intensive care services. This contains broad guidance for planning but does not define how delirium should be prevented or managed, or provide detailed descriptions of the interventions. The UK Intensive Care Society's *Guidance for delirium in the critically ill patient* [55] was generated by expert opinion after evidence review. This does not define a care package, but the principles of ICU delirium identification, prevention and management. The US Society of Critical Care Medicine Pain, Agitation, Delirium, Immobility and Sleep defines 37 questions prioritised by patients, researchers and clinicians and provides evidence review and a summary recommendation for each [31]. These have been operationalised into the ABCDEF bundle to aid in their implementation. The ABCDEF bundle is to: Assess, prevent and manage pain; Both spontaneous awakening and breathing trials; Choice of analgesia and sedation; Delirium monitoring and management; Early exercise/mobility; and Family engagement [77].

Multiple randomised studies have shown that out of all strategies, non‐pharmacological multifaceted bundles are the most promising. These lead to a significant reduction of the occurrence of delirium while also shortening duration of ventilator days and improving functional independence [78]. The Society of Critical Care Medicine has launched the ICU Liberation Campaign that provides evidence‐based guidance on how to implement its Pain, Agitation, Delirium, Immobility and Sleep guidelines and the ABCDEF bundle in ICUs [79]. A study in 15,000 patients from 68 diverse ICUs showed that implementation of the ABCDEF bundle reduced coma; delirium; and the use of physical restraint. It also reduced time on a ventilator; increased survival; avoided ICU readmission; and increased the chance of being discharged home [80].

However, the sustainable implementation of these guidelines has proved challenging across different countries and healthcare systems [80, 81, 82]. Over the past two decades, there has been minimal evolution in the recommendations themselves [83], paralleled by limited progress in their consistent implementation in practice [84]. Facilitators to embed high‐quality delirium care in the ICU that are highlighted consistently include strong clinical leadership; interdisciplinary collaboration; supportive organisational culture; and audit and feedback [85]. Barriers vary by setting but commonly include the low prioritisation of delirium within the ICU; the absence of structured delirium care protocols; limitations in physical space or resources; loss of experienced staff familiar with ICU delirium management; and changes in the culture of ICU nursing [85]. Complex multicomponent interventions are especially vulnerable to poor multidisciplinary co‐ordination. These care bundles also lack the systems and processes needed alongside them to convert the multiple components into consistent, routine practice [86, 87]. Sustainable adopting of evidence‐based guidelines will likely require implementation science approaches including behaviour science informed, system‐level strategies co‐designed by patients and multidisciplinary healthcare providers. This bundle could then be tested in an appropriately designed clinical trial to evaluate whether it reduced delirium incidence and or duration.

### Follow up

Follow up services after critical care are offered in most ICUs. The resources, staffing and service provision are highly variable and limited. Most follow up services are made up of a wider multidisciplinary team including at least two healthcare professionals such as a critical care physician; a critical care nurse; occupational therapists; physiotherapists; and, in < 50%, a clinical psychologist [86]. However, the best clinical practice model of ICU follow‐up service has not been fully defined.

Whilst guidance suggests an ICU follow‐up appointment 2–3 months after hospital discharge [87], it is understood widely that physical and psychological recovery after critical care (with or without delirium) can take many months if not years [88]. Given limited resources, only a small number of patients can be seen in clinic. This is important as delirium is still missed on discharge letters from hospitals, leaving many patients and their families grappling with the long‐term consequences, with sometimes haunting flashbacks and memories of their ICU experience [89]. Patient perception of reality and their own behaviour while delirious are often associated with shame and fear and therefore rarely talked about, making the follow‐up service often the only outlet families and patients have to talk about their experience in a safe, supportive setting with an expert team [90, 91].

Whilst readiness for a visit to the ICU needs to be assessed and planned carefully, the experience of coming back to the ICU can be helpful. Seeing the room where they were cared for, and revisiting the ICU environment and staff, can dissipate some of the real or frightening memories they experienced while delirious [92]. Diaries kept by staff and/or family members have been shown to be another beneficial tool for patients, reassuring them that events did not occur as perceived while delirious [93, 94]. Going through timelines, explaining what delirium is and how it manifests are simple interventions to aid patient and family understanding of their ICU journey.

There is little known about the ongoing support needs of ICU survivors with delirium and their carers or the support currently available after discharge from hospital. To our knowledge, no studies have examined longer‐term recovery after delirium. There is now a growing number of delirium‐specific follow‐up services emerging all over the UK [84]. A pilot study is currently under way to assess the longer‐term recovery needs for patients after delirium (non‐ICU specific) and this will hopefully be a significant step to further guide long‐term patient management.

## Conclusion

ICU delirium is common and now recognised as a serious clinical issue with long term consequences. In many cases delirium can be preventable. While assessment and diagnosis have improved over the last few years, prevention and treatment strategies require the involvement of the entire multidisciplinary team on an ICU. The implementation of non‐pharmacological care bundles is likely to be the most effective prevention and management strategy. Integrating these in a behavioural intervention with appropriate resources is likely to be the best way to prevent and manage delirium in the ICU. Further research is needed to understand the needs of patients and their families/carers after hospital discharge.

## References

[1] Salluh JI , Soares M , Teles JM , et al. Delirium epidemiology in critical care (DECCA): an international study. Crit Care 2010; 14: R210. 10.1186/cc9333. 21092264 PMC3220001

[2] American Psychiatric Association . Diagnostic and Statistical Manual of Mental Disorders (DSM‐5®), Fifth Edition: American Psychiatric Pub, 2013.

[3] Hughes CG , Hayhurst CJ , Pandharipande PP , et al. Association of delirium during critical illness with mortality: multicenter prospective cohort study. Anesth Analg 2021; 133: 1152–1161. 10.1213/ane.0000000000005544. 33929361 PMC8542584

[4] Klein Klouwenberg PM , Zaal IJ , Spitoni C , et al. The attributable mortality of delirium in critically ill patients: prospective cohort study. BMJ 2014; 349: g6652. 10.1136/bmj.g6652. 25422275 PMC4243039

[5] Salluh JIF , Wang H , Schneider EB , et al. Outcome of delirium in critically ill patients: systematic review and meta‐analysis. BMJ 2015; 350: h2538. 10.1136/bmj.h2538. 26041151 PMC4454920

[6] Shehabi Y , Riker RR , Bokesch PM , Wisemandle W , Shintani A , Ely EW , SEDCOM (Safety and Efficacy of Dexmedetomidine Compared With Midazolam) Study Group . Delirium duration and mortality in lightly sedated, mechanically ventilated intensive care patients. Crit Care Med 2010; 38: 2311–2318. 10.1097/CCM.0b013e3181f85759. 20838332

[7] Patel SB , Poston JT , Pohlman A , Hall JB , Kress JP . Rapidly reversible, sedation‐related delirium versus persistent delirium in the intensive care unit. Am J Respir Crit Care Med 2014; 189: 658–665. 10.1164/rccm.201310-1815OC. 24423152

[8] Fiest KM , Soo A , Hee Lee C , Niven DJ , Ely EW , Doig CJ , Stelfox HT . Long‐term outcomes in ICU patients with delirium: a population‐based cohort study. Am J Respir Crit Care Med 2021; 204: 412–420. 10.1164/rccm.202002-0320OC. 33823122 PMC8480248

[9] Pandharipande PP , Girard TD , Jackson JC , et al. Long‐term cognitive impairment after critical illness. N Engl J Med 2013; 369: 1306–1316. 10.1056/NEJMoa1301372. 24088092 PMC3922401

[10] Brown KN , Soo A , Faris P , Patten SB , Fiest KM , Stelfox HT . Association between delirium in the intensive care unit and subsequent neuropsychiatric disorders. Crit Care 2020; 24: 476. 10.1186/s13054-020-03193-x. 32736572 PMC7393876

[11] Rabiee A , Nikayin S , Hashem MD , et al. Depressive symptoms after critical illness: a systematic review and meta‐analysis. Crit Care Med 2016; 44: 1744–1753. 10.1097/ccm.0000000000001811. 27153046 PMC7418220

[12] Unoki T , Sakuramoto H , Uemura S , et al. Prevalence of and risk factors for post‐intensive care syndrome: multicenter study of patients living at home after treatment in 12 Japanese intensive care units, SMAP‐HoPe study. PLoS One 2021; 16: e0252167. 10.1371/journal.pone.0252167. 34043682 PMC8158919

[13] Wolters AE , Peelen LM , Welling MC , et al. Long‐term mental health problems after delirium in the ICU. Crit Care Med 2016; 44: 1808–1813. 10.1097/ccm.0000000000001861. 27513540

[14] Vidal EI , Villas Boas PJ , Valle AP , Cerqueira AT , Fukushima FB . Delirium in older adults. BMJ 2013; 346: f2031. 10.1136/bmj.f2031. 23571740

[15] Jones KL , Kundakci B , Booth A , Falzon L , Gibbison B , Pufulete M . A systematic meta‐review of interventions to prevent and manage delirium in the intensive care unit: part 1 – pharmacological interventions. Crit Care 2025; 29: 540. 10.1186/s13054-025-05615-0. 41469920 PMC12751364

[16] Jones KL , Kundakci B , Booth A , Pufulete M , Gibbison B . Protocol for a meta‐review of interventions to prevent and manage ICU delirium. BMJ Open 2025; 15: e090815. 10.1136/bmjopen-2024-090815. PMC11815468 39933812

[17] Kundakci B , Jones K , Booth A , Falzon L , Pufulete M , Gibbison B . A systematic meta‐review of interventions to prevent and manage delirium in the intensive care unit: part 2 – non‐pharmacological and multicomponent interventions. Crit Care 2025; 29: 501. 10.1186/s13054-025-05726-8. 41272698 PMC12639998

[18] Kundakci B , Jones KL , Booth A , Parslow RM , Moore AJ , Gibbison B , Pufulete M . Factors contributing to the implementation of interventions to prevent and manage intensive care unit delirium: a systematic review protocol. BMJ Open 2025; 15: e093338. 10.1136/bmjopen-2024-093338. PMC12039013 40295133

[19] Peterson JF , Pun BT , Dittus RS , Thomason JW , Jackson JC , Shintani AK , Ely EW . Delirium and its motoric subtypes: a study of 614 critically ill patients. J Am Geriatr Soc 2006; 54: 479–484. 10.1111/j.1532-5415.2005.00621.x. 16551316

[20] Krewulak KD , Stelfox HT , Leigh JP , Ely EW , Fiest KM . Incidence and prevalence of delirium subtypes in an adult ICU: a systematic review and meta‐analysis. Crit Care Med 2018; 46: 2029–2035. 10.1097/ccm.0000000000003402. 30234569

[21] Bowman EML , Cunningham EL , Page VJ , McAuley DF . Phenotypes and subphenotypes of delirium: a review of current categorisations and suggestions for progression. Crit Care 2021; 25: 334. 10.1186/s13054-021-03752-w. 34526093 PMC8441952

[22] la Cour KN , Andersen‐Ranberg NC , Weihe S , et al. Distribution of delirium motor subtypes in the intensive care unit: a systematic scoping review. Crit Care 2022; 26: 53. 10.1186/s13054-022-03931-3. 35241132 PMC8896322

[23] Bradley J , Tang F , Panzarella Z , Nanney J , Hammel I , Bryant B , Cole D . Persistent inpatient delirium associated with increased length of stay and mortality. PLoS One 2025; 20: e0331245. 10.1371/journal.pone.0331245. 40892851 PMC12404460

[24] Li H‐C , Yeh TY‐C , Wei Y‐C , et al. Association of incident delirium with short‐term mortality in adults with critical illness receiving mechanical ventilation. JAMA Netw Open 2022; 5: e2235339. 10.1001/jamanetworkopen.2022.35339. 36205994 PMC9547314

[25] Siddiqi N , House AO , Holmes JD . Occurrence and outcome of delirium in medical in‐patients: a systematic literature review. Age Ageing 2006; 35: 350–364. 10.1093/ageing/afl005. 16648149

[26] van den Boogaard M , Peters SA , van der Hoeven JG , Dagnelie PC , Leffers P , Pickkers P , Schoonhoven L . The impact of delirium on the prediction of in‐hospital mortality in intensive care patients. Crit Care 2010; 14: R146. 10.1186/cc9214. 20682037 PMC2945129

[27] Zhang Z , Pan L , Ni H . Impact of delirium on clinical outcome in critically ill patients: a meta‐analysis. Gen Hosp Psychiatry 2013; 35: 105–111. 10.1016/j.genhosppsych.2012.11.003. 23218845

[28] Ely EW , Gautam S , Margolin R , et al. The impact of delirium in the intensive care unit on hospital length of stay. Intensive Care Med 2001; 27: 1892–1900. 10.1007/s00134-001-1132-2. 11797025 PMC7095464

[29] Girard TD , Thompson JL , Pandharipande PP , et al. Clinical phenotypes of delirium during critical illness and severity of subsequent long‐term cognitive impairment: a prospective cohort study. Lancet Respir Med 2018; 6: 213–222. 10.1016/S2213-2600(18)30062-6. 29508705 PMC6709878

[30] Pisani MA , Kong SY , Kasl SV , Murphy TE , Araujo KL , Van Ness PH . Days of delirium are associated with 1‐year mortality in an older intensive care unit population. Am J Respir Crit Care Med 2009; 180: 1092–1097. 10.1164/rccm.200904-0537OC. 19745202 PMC2784414

[31] Devlin JW , Skrobik Y , Gelinas C , et al. Clinical practice guidelines for the prevention and management of pain, agitation/sedation, delirium, immobility, and sleep disruption in adult patients in the ICU. Crit Care Med 2018; 46: e825–e873. 10.1097/ccm.0000000000003299. 30113379

[32] Ely EW , Shintani A , Truman B , et al. Delirium as a predictor of mortality in mechanically ventilated patients in the intensive care unit. JAMA 2004; 291: 1753–1762. 10.1001/jama.291.14.1753. 15082703

[33] Wolters AE , van Dijk D , Pasma W , et al. Long‐term outcome of delirium during intensive care unit stay in survivors of critical illness: a prospective cohort study. Crit Care 2014; 18: R125. 10.1186/cc13929. 24942154 PMC4095683

[34] Girard TD , Jackson JC , Pandharipande PP , et al. Delirium as a predictor of long‐term cognitive impairment in survivors of critical illness. Crit Care Med 2010; 38: 1513–1520. 10.1097/CCM.0b013e3181e47be1. 20473145 PMC3638813

[35] Inouye SK , Westendorp RG , Saczynski JS . Delirium in elderly people. Lancet 2014; 383: 911–922. 10.1016/s0140-6736(13)60688-1. 23992774 PMC4120864

[36] van Gool WA , van de Beek D , Eikelenboom P . Systemic infection and delirium: when cytokines and acetylcholine collide. Lancet 2010; 375: 773–775. 10.1016/S0140-6736(09)61158-2. 20189029

[37] Haggstrom L , Welschinger R , Caplan GA . Functional neuroimaging offers insights into delirium pathophysiology: a systematic review. Australas J Ageing 2017; 36: 186–192. 10.1111/ajag.12417. 28519903

[38] Nitchingham A , Kumar V , Shenkin S , Ferguson KJ , Caplan GA . A systematic review of neuroimaging in delirium: predictors, correlates and consequences. Int J Geriatr Psychiatry 2018; 33: 1458–1478. 10.1002/gps.4724. 28574155

[39] Numan T , Slooter AJC , van der Kooi AW , Hoekman AML , Suyker WJL , Stam CJ , van Dellen E . Functional connectivity and network analysis during hypoactive delirium and recovery from anesthesia. Clin Neurophysiol 2017; 128: 914–924. 10.1016/j.clinph.2017.02.022. 28402867

[40] Gunther ML , Morandi A , Krauskopf E , et al. The association between brain volumes, delirium duration, and cognitive outcomes in intensive care unit survivors: the VISIONS cohort magnetic resonance imaging study. Crit Care Med 2012; 40: 2022–2032. 10.1097/CCM.0b013e318250acc0. 22710202 PMC3697780

[41] Hayhurst CJ , Pandharipande PP , Hughes CG . Intensive care unit delirium: a review of diagnosis, prevention, and treatment. Anesthesiology 2016; 125: 1229–1241. 10.1097/ALN.0000000000001378. 27748656 PMC5119532

[42] Zaal IJ , Devlin JW , Peelen LM , Slooter AJC . A systematic review of risk factors for delirium in the ICU. Crit Care Med 2015; 43: 40–47. 10.1097/CCM.0000000000000625. 25251759

[43] Lobo‐Valbuena B , Gordo F , Abella A , et al. Risk factors associated with the development of delirium in general ICU patients. A prospective observational study. PLoS One 2021; 16: e0255522. 10.1371/journal.pone.0255522. 34473734 PMC8412262

[44] Ely EW , Girard TD , Shintani AK , et al. Apolipoprotein e4 polymorphism as a genetic predisposition to delirium in critically ill patients. Crit Care Med 2007; 35: 112–117. 10.1097/01.Ccm.0000251925.18961.Ca. 17133176

[45] van Munster BC , Korevaar JC , Zwinderman AH , Leeflang MM , de Rooij SE . The association between delirium and the apolipoprotein e epsilon 4 allele: new study results and a meta‐analysis. Am J Geriatr Psychiatry 2009; 17: 856–862. 10.1097/JGP.0b013e3181ab8c84. 19910874

[46] van Gelder TG , van Diem‐Zaal IJ , Dijkstra‐Kersten SMA , de Mul N , Lalmohamed A , Slooter AJC . The risk of delirium after sedation with propofol or midazolam in intensive care unit patients. Br J Clin Pharmacol 2024; 90: 1471–1479. 10.1111/bcp.16031. 38482541

[47] Lightman SL , Conway‐Campbell BL . Circadian and ultradian rhythms: clinical implications. J Intern Med 2024; 296: 121–138. 10.1111/joim.13795. 38825772

[48] Gibbison B , Keenan DM , Roelfsema F , et al. Dynamic pituitary‐adrenal interactions in the critically ill after cardiac surgery. J Clin Endocrinol Metab 2020; 105: 1327–1342. 10.1210/clinem/dgz206. 31738827 PMC7089849

[49] Hughes CG , Boncyk CS , Fedeles B , et al. Association between cholinesterase activity and critical illness brain dysfunction. Crit Care 2022; 26: 377. 10.1186/s13054-022-04260-1. 36474266 PMC9724294

[50] Iyo M , Namba H , Fukushi K , et al. Measurement of acetylcholinesterase by positron emission tomography in the brains of healthy controls and patients with Alzheimer's disease. Lancet 1997; 349: 1805–1809. 10.1016/S0140-6736(96)09124-6. 9269216

[51] Vondeling AM , Knol W , Egberts TCG , Slooter AJC . Anticholinergic drug exposure at intensive care unit admission affects the occurrence of delirium. A prospective cohort study. Eur J Intern Med 2020; 78: 121–126. 10.1016/j.ejim.2020.04.062. 32487370

[52] Reisinger M , Reininghaus EZ , Biasi J , Fellendorf FT , Schoberer D . Delirium‐associated medication in people at risk: a systematic update review, meta‐analyses, and grade‐profiles. Acta Psychiatr Scand 2023; 147: 16–42. 10.1111/acps.13505. 36168988 PMC10092229

[53] Duprey MS , Dijkstra‐Kersten SMA , Zaal IJ , et al. Opioid use increases the risk of delirium in critically ill adults independently of pain. Am J Respir Crit Care Med 2021; 204: 566–572. 10.1164/rccm.202010-3794OC. 33835902 PMC8491270

[54] Ely EW , Inouye SK , Bernard GR , et al. Delirium in mechanically ventilated patients: validity and reliability of the confusion assessment method for the intensive care unit (CAM‐ICU). JAMA 2001; 286: 2703–2710. 10.1001/jama.286.21.2703. 11730446

[55] Intensive Care Society . Guidance for delirium in the critically ill patient. 2025. https://ics.ac.uk/resource/guidance‐for‐delirium‐in‐the‐critically‐ill‐patient‐pdf.html (accessed 10/02/2026).

[56] Bergeron N , Dubois MJ , Dumont M , Dial S , Skrobik Y . Intensive care delirium screening checklist: evaluation of a new screening tool. Intensive Care Med 2001; 27: 859–864. 11430542 10.1007/s001340100909

[57] Bellelli G , Morandi A , Davis DH , et al. Validation of the 4AT, a new instrument for rapid delirium screening: a study in 234 hospitalised older people. Age Ageing 2014; 43: 496–502. 10.1093/ageing/afu021. 24590568 PMC4066613

[58] Chanques G , Ely EW , Garnier O , et al. The 2014 updated version of the confusion assessment method for the intensive care unit compared to the 5th version of the diagnostic and statistical manual of mental disorders and other current methods used by intensivists. Ann Intensive Care 2018; 8: 33. 10.1186/s13613-018-0377-7. 29492696 PMC5833335

[59] Khan BA , Perkins AJ , Gao S , et al. The confusion assessment method for the ICU‐7 delirium severity scale: a novel delirium severity instrument for use in the ICU. Crit Care Med 2017; 45: 851–857. 10.1097/ccm.0000000000002368. 28263192 PMC5392153

[60] Lewis K , Alshamsi F , Carayannopoulos KL , et al. Dexmedetomidine vs other sedatives in critically ill mechanically ventilated adults: a systematic review and meta‐analysis of randomized trials. Intensive Care Med 2022; 48: 811–840. 10.1007/s00134-022-06712-2. 35648198

[61] Shehabi Y , Howe BD , Bellomo R , et al. Early sedation with dexmedetomidine in critically ill patients. N Engl J Med 2019; 380: 2506–2517. 10.1056/NEJMoa1904710. 31112380

[62] Walsh TS , Parker RA , Aitken LM , et al. Dexmedetomidine‐ or clonidine‐based sedation compared with propofol in critically ill patients: the a2b randomized clinical trial. JAMA 2025; 334: 32–45. 10.1001/jama.2025.7200. 40388916 PMC12090071

[63] Shehabi Y , Serpa Neto A , Bellomo R , et al. Dexmedetomidine and propofol sedation in critically ill patients and dose‐associated 90‐day mortality: a secondary cohort analysis of a randomized controlled trial (spice iii). Am J Respir Crit Care Med 2023; 207: 876–886. 10.1164/rccm.202206-1208OC. 36215171

[64] Hughes CG , Girard TD , Pandharipande PP . Daily sedation interruption versus targeted light sedation strategies in ICU patients. Crit Care Med 2013; 41: S39–S45. 10.1097/CCM.0b013e3182a168c5. 23989094

[65] Burry LD , Cheng W , Williamson DR , et al. Pharmacological and non‐pharmacological interventions to prevent delirium in critically ill patients: a systematic review and network meta‐analysis. Intensive Care Med 2021; 47: 943–960. 10.1007/s00134-021-06490-3. 34379152 PMC8356549

[66] Cruccioli MM , Carneiro PHT , Pitanga JFJ , et al. Haloperidol versus atypical antipsychotics for delirium in hospitalized and ICU patients: a systematic review and meta‐analysis. Clin Neuropharmacol 2025; 48: 145–150. 10.1097/WNF.0000000000000650. 40939180

[67] Zakhary T , Ahmed I , Luttfi I , Montasser M . Quetiapine versus haloperidol in the management of hyperactive delirium: randomized controlled trial. Neurocrit Care 2024; 41: 550–557. 10.1007/s12028-024-01948-w. 38561588 PMC11377686

[68] Kamdar BB , King LM , Collop NA , et al. The effect of a quality improvement intervention on perceived sleep quality and cognition in a medical ICU. Crit Care Med 2013; 41: 800–809. 10.1097/CCM.0b013e3182746442. 23314584 PMC3579000

[69] Kamdar BB , Needham DM , Collop NA . Sleep deprivation in critical illness: its role in physical and psychological recovery. J Intensive Care Med 2012; 27: 97–111. 10.1177/0885066610394322. 21220271 PMC3299928

[70] Schreiber MP , Colantuoni E , Bienvenu OJ , et al. Corticosteroids and transition to delirium in patients with acute lung injury. Crit Care Med 2014; 42: 1480–1486. 10.1097/ccm.0000000000000247. 24589640 PMC4028387

[71] Meduri GU , Shih M‐C , Bridges L , et al. Low‐dose methylprednisolone treatment in critically ill patients with severe community‐acquired pneumonia. Intensive Care Med 2022; 48: 1009–1023. 10.1007/s00134-022-06684-3. 35723686 PMC9208259

[72] Tongyoo S , Permpikul C , Mongkolpun W , Vattanavanit V , Udompanturak S , Kocak M , Meduri GU . Hydrocortisone treatment in early sepsis‐associated acute respiratory distress syndrome: results of a randomized controlled trial. Crit Care 2016; 20: 329. 10.1186/s13054-016-1511-2. 27741949 PMC5065699

[73] Burry L , Hutton B , Williamson DR , et al. Pharmacological interventions for the treatment of delirium in critically ill adults. Cochrane Database Syst Rev 2019; 2019: CD011749. 10.1002/14651858.CD011749.pub2. PMC6719921 31479532

[74] van Eijk MM , Roes KC , Honing ML , et al. Effect of rivastigmine as an adjunct to usual care with haloperidol on duration of delirium and mortality in critically ill patients: a multicentre, double‐blind, placebo‐controlled randomised trial. Lancet 2010; 376: 1829–1837. 10.1016/s0140-6736(10)61855-7. 21056464

[75] National Institute for Health and Care Excellence . Delirium: Prevention, diagnosis and management in hospital and long‐term care [CG103]. 2023. https://www.nice.org.uk/guidance/cg103 (accessed 10/02/2026). 31971702

[76] Faculty of Intensive Care Medicine . Guidelines for the provision of intensive care services v2.1. 2022. https://www.ficm.ac.uk/standards/guidelines‐for‐the‐provision‐of‐intensive‐care‐services (accessed 10/02/2026).

[77] Marra A , Ely EW , Pandharipande PP , Patel MB . The ABCDEF bundle in critical care. Crit Care Clin 2017; 33: 225–243. 10.1016/j.ccc.2016.12.005. 28284292 PMC5351776

[78] Barr J , Downs B , Ferrell K , et al. Improving outcomes in mechanically ventilated adult ICU patients following implementation of the ICU liberation (ABCDEF) bundle across a large healthcare system. Crit Care Explor 2024; 6: e1001. 10.1097/cce.0000000000001001. 38250248 PMC10798758

[79] Ely EW . The ABCDEF bundle: science and philosophy of how ICU liberation serves patients and families. Crit Care Med 2017; 45: 321–330. 10.1097/ccm.0000000000002175. 28098628 PMC5830123

[80] Pun BT , Balas MC , Barnes‐Daly MA , et al. Caring for critically ill patients with the ABCDEF bundle: results of the ICU liberation collaborative in over 15,000 adults. Crit Care Med 2019; 47: 3–14. 10.1097/ccm.0000000000003482. 30339549 PMC6298815

[81] Trogrlić Z , van der Jagt M , Bakker J , et al. Implementation of the ABCDE bundle in intensive care units: a multicenter prospective study. Crit Care 2020; 24: 418. 10.1186/s13054-020-03175-1.32653015

[82] Vincelette C , D'Aragon F , Stevens L‐M , Rochefort CM . The characteristics and factors associated with omitted nursing care in the intensive care unit: A cross‐sectional study. Intensive Crit Care Nurs 2023; 75: 103343. 10.1016/j.iccn.2022.103343. 36371393

[83] Intensive Care Society . Detection, prevention and treatment of delirium in critically ill patients. 2006. https://icmwk.com/wp‐content/uploads/2014/02/ukcpa_delirium_2006.pdf (accessed 10/02/2026).

[84] Gibbison B , Johnson AF , Rowan KM , et al. Delirium identification, prevention and management in intensive care units in England, Wales and Northern Ireland: a survey of practice. Anaesthesia 2026; 81: 41–50. 10.1111/anae.16728. 40790899 PMC12747587

[85] Parslow RM , Gibbison B , Pufulete M , Rowan KM , Moore AJ . Barriers and facilitators to the delivery of delirium care in intensive care units: an analysis informed by the theoretical domains framework. Anaesthesia 2026; 81: 213–221. 10.1111/anae.70017. 41055047 PMC12803597

[86] Connolly B , Milton‐Cole R , Adams C , et al. Recovery, rehabilitation and follow‐up services following critical illness: an updated UK national cross‐sectional survey and progress report. BMJ Open 2021; 11: e052214. 10.1136/bmjopen-2021-052214. PMC8491421 34607869

[87] National Institute for Health and Care Excellence . Rehabilitation after critical illness in adults [CG83]. 2009. https://www.nice.org.uk/guidance/cg83 (accessed 10/02/2026). 39480975

[88] Faculty of Intensive Care Medicine . Life after critical illness. https://www.ficm.ac.uk/criticalfutures/life‐after‐critical‐illness (accessed 10/02/2026).

[89] Ibitoye T , So S , Shenkin SD , et al. Delirium is under‐reported in discharge summaries and in hospital administrative systems: a systematic review. Delirium 2023; 2023: 74541. 10.56392/001c.74541. 39654697 PMC7617113

[90] Pollard C , Fitzgerald M , Ford K . Delirium: the lived experience of older people who are delirious post‐orthopaedic surgery. Int J Ment Health Nurs 2015; 24: 213–221. 10.1111/inm.12132. 25976839

[91] Van Rompaey B , Van Hoof A , van Bogaert P , Timmermans O , Dilles T . The patient's perception of a delirium: a qualitative research in a Belgian intensive care unit. Intensive Crit Care Nurs 2016; 32: 66–74. 10.1016/j.iccn.2015.03.002. 26612360

[92] Engstrom A , Andersson S , Soderberg S . Re‐visiting the ICU experiences of follow‐up visits to an ICU after discharge: a qualitative study. Intensive Crit Care Nurs 2008; 24: 233–241. 10.1016/j.iccn.2008.03.002. 18434156

[93] Barreto BB , Luz M , Rios MNO , Lopes AA , Gusmao‐Flores D . The impact of intensive care unit diaries on patients' and relatives' outcomes: a systematic review and meta‐analysis. Crit Care 2019; 23: 411. 10.1186/s13054-019-2678-0. 31842929 PMC6916011

[94] Bosco V , Mercuri C , Giordano V , et al. Enhancing ICU care with nurse‐written diaries. Nurs Crit Care 2024; 29: 1355–1362. 10.1111/nicc.13161. 39308054

